# Male breast cancer: a 32-year retrospective analysis in radiation therapy referral center in northern Iran

**DOI:** 10.1097/MS9.0000000000002571

**Published:** 2024-09-12

**Authors:** Mahboobeh Asgharian, Dariush Moslemi, Hossein-Ali Nikbakht, Mohammad-Ali Jahani, Ali Bijani, Hakimeh Mehdizadeh

**Affiliations:** a Student Research Committee, Babol Medical University of Medical Sciences; b Department of Radiation Oncology, Babol University of Medical Sciences; c Social Determinants of Health Research Center, Health Research Institute, Babol University of Medical Sciences; d Social Determinants of Health Research Center, Health Research Institute, Babol University of Medical Sciences; e Babol University of Medical Sciences, Babol, I R Iran

**Keywords:** breast neoplasms, male, mastectomy, metastasis, risk factor

## Abstract

**Background:**

Breast cancer commonly occurs in women, and male breast cancer makes up less than 1% of all cases of breast cancer. The limited prevalence of male breast cancer has led to decreased attention being paid to this condition, resulting in its diagnosis occurring at later ages and at more severe disease stages.

**Objectives:**

This study evaluates the demographic and clinicopathological characteristics of male patients diagnosed with breast cancer who visited the northern region of Iran from 1992 to 2023.

**Methods:**

This descriptive study reviewed data from 58 cases of male breast cancer between 1992 and 2023. The study aimed to examine and describe the information connected to these patients. The data were analyzed with SPSS.22 set at *P* value less than 0.05.

**Results:**

The mean age of the patients examined was 62.10±13.40 years, while their mean BMI was 27.08±4.95. The study found no statistically significant correlation between BMI with stage and kinds of recurrence, including metastasis and local recurrence (*P*>0.05). The right and left breast involvement rates were equal (48.28%) in 28 cases, and 2 cases (3.40%) had bilateral involvement. The bone was the predominant site of metastasis, accounting for 69.23% of cases. The analysis revealed no significant correlation between stage and metastasis (*P*=1.000) or local recurrence (*P*=0.543). Most metastasis and all the local recurrence were observed in stages 3 and 4.

**Conclusion:**

Male breast cancer mainly occurs in older age and is diagnosed in the advanced stages of the disease. Therefore, it is recommended to inform men and develop suitable screening programs, especially in high-risk families.

## Introduction

Highlights

- There has been a noticeable rise in the occurrence of male breast cancer in recent years, with an increase of 20–25% in the past few decades.
- Male breast cancer accounts for less than 1% of all breast cancers, which has increased in recent decades and has a 60% higher mortality rate than female breast cancer.
- The treatment of breast cancer in men is based on studies of female breast cancer, while men have different biological and molecular triggers compared to women.
- The common sites of metastasis were bone, brain, lung, and liver, respectively.
- The majority of metastasis and all the local recurrence were observed in stages 3 and 4.

One of the most common malignancies known in women is breast cancer^1^. However, with a much lower prevalence, it also occurs in men^2^, accounting for only 0.5% of all malignancies detected in men^3,4^. There has been a noticeable rise in the occurrence of male breast cancer in recent years, with an increase of 20–25% in the past few decades^5,6^. Male breast cancer accounts for less than 1% of all breast cancers, which has increased in recent decades and has a 60% higher mortality rate than female breast cancer^7^.

Family history, ageing, hormonal disorders, and radiation exposure are among the main risk factors of breast cancer in both sexes^8^. Increased BMI and abdominal obesity are risk factors for breast cancer in men, independently of obesity alone^9^, The prevalence of obesity seems to be one of the reasons for the increase in breast cancer in men in developing countries^10–12^. Although male breast cancer has some characteristics similar to female breast cancer, they have significant differences in presentation, epidemiology and prognosis^13^. Men’s breast cancer is usually diagnosed in more advanced stages and at older ages than women. In addition, men have a lower survival rate after diagnosis than women^14,15^. Currently, the survival rate of men with breast cancer is 14.6% lower than that of women^16^. Also, Estrogen receptors (ER) and progesterone receptors (PR) are more positive in men than in women. Human Epidermal growth factor Receptor-2 (HER-2) is less expressed, and men with positive ER and PR showed better survival^17^. Distant metastasis is a significant determinant of outcome in breast cancer^18^, which occurs in males about 7% more than females^19^. The treatment of breast cancer in men is based on studies of female breast cancer, while men have different biological and molecular triggers compared to women^20^. It seems that in terms of etiology and epidemiology, there are differences in men compared to women

The prevalence of breast cancer in men in Iran rises with age and particularly peaks in men aged 80 and above^21^. Geographical and socio-economic inequalities in the incidence and mortality of women’s breast cancer may have an impact on Iran’s population^22^. Research on male breast cancer has been carried out in Iran and several other countries. In a study in Shiraz, located in the southern region of Iran, investigating 58 cases of male breast cancer, the average age of male breast cancer patients was 60 years. Additionally, they detected a rising trend in the incidence rate of male breast cancer^23^. In a study, stated that the older age of men at the time of diagnosis, HER_2 positive and metastasis to other organs reduced the survival rate^24^. A study in Pakistan showed that the average age of the disease was 63 years, and most patients (36.8%) were in stage 3. The 5-year overall survival was 38.2%, the average survival was 36 months, and the 5-year disease-free survival (DFS) was 33.7%^25^. In the study of Cardoso and colleagues, it was shown that men are usually ER and PR positive and HER_2 negative. Although 56% of patients had T1 tumors, only 4% of patients underwent breast conserving surgery (BCS), and the most common surgical procedure performed on men was modified radical mastectomy (MRM)^26^. A study conducted in Turkey investigated the prognosis of male patients with metastatic breast cancer. The findings showed that brain metastasis, the number of metastatic sites, and a history of alcohol intake were identified as poor prognostic factors in men. The most common sites of metastasis in men were bone (75%), lung (39.3%), and brain (21.4%), respectively^27^.

Considering that so limited studies have been done on male breast cancer and there is not enough awareness in men, also community awareness and screening programs are conducted with a focus on women^28^, It is necessary to conduct more studies on breast cancer in men and its risk factors, and to raise awareness in both sexes effectively. Also, men in high-risk families should be screened, monitored and managed just like women. Despite the studies, detailed information about the biological characteristics of this disease and its specific risk factors and prognostic factors is limited. Most of the available information is based on studies on this disease in women. Therefore, it is necessary to conduct an extensive study on male breast cancer, especially in Iran. The present study, by examining the demographic and clinical characteristics of male breast cancer patients in the north of Iran over f almost 30 years, can contribute to other epidemiological and population-based studies. Therefore, the present study aimed to evaluate all male patients with breast cancer (32-year retrospective analysis) in a radiation therapy referral center in northern Iran between 1992 and 2023.

## Methods

This study is a retrospective descriptive and analytical research conducted in 2023, following ethical guidelines.

### Study design

The research population was all male patients with breast cancer referred from 1992 to 2023, and finally, 58 patients were included in the study by census.

### Data collection and variables

The inclusion criteria for the study were all men diagnosed with breast cancer who were referred to that center between 1992 and 2023, and the exclusion criteria were the patients with duplicate records deleted. It is based on the information registered in the patient’s file and the hospital’s information system, which is the criteria for breast cancer diagnosis, biopsy and pathology confirmation. A total of 58 patients were referred during these years. Due to the impossibility of contacting the patients and their families in older years (due to the death of the patients, after not going to the hospital for further treatment and following up with the hospital staff or lack of appropriate contact numbers), the data recorded in the patient’s medical records were analyzed. However, patients with incomplete records were also included in the study. A checklist was prepared for data collection, which included information such as age, marital status, place of residence, patients’ BMI, type of surgery, receptor status, metastasis, disease stage and local recurrence, which were extracted from the physical and electronic records of the hospital. To record data such as the stage of the disease (Recorded in patient files), local recurrence, and metastasis, we referred to the hospital’s medical records archive. This data are based on the information registered in the patient’s file and the hospital’s information system, which includes the criteria for breast cancer diagnosis, biopsy, and pathology confirmation. The work has been reported in line with the Strengthening the Reporting of Cohort Studies in Surgery criteria^29^.

### Statistical analysis

The study provided statistical indices to describe the characteristics of patients. Descriptive characteristics of patients were reported with statistical indicators for quantitative variables with the default of normality using the Measures of Central Tendency of Mean (S.D), median (interquartile range), minimum, maximum and frequency (percentage) for qualitative variables. In order to check the related statistical tests, the normality of the data was first checked using the Kolmogorov–Smirnov test. The χ^2^ test was used to evaluate the significant relationship between the qualitative variables., In contrast, Fisher’s exact test was utilized in cases with limitations in the predicted frequency. The independent *t*-test was employed to examine the correlation between quantitative variables in two separate scenarios, and in cases where normality was absent, the Mann–Whitney test was utilized. The analyses were conducted with SPSS22, and a significance threshold of *P* less than 0.05 was used.

### Ethics approval

This study was carried out after the approval of the ethics committee of the University of Medical Sciences in such a way that all steps of the Declaration of Helsinki were observed.

## Results

In this study, 58 male patients with breast cancer were investigated ; according to the results of Table 1, 35 (60.34%) patients were over 60 years old. 57 (98.28%) patients were married, and 43 (74.14%) patients were from the urban population. (Table 1).

**Table 1:** Demographic characteristics of cases with breast cancer (1992–2023)

Variable	Frequency (total:58) (percentage), *n* (%)
Age	
<50	11 (18.97)
50–59	12 (20.69)
60–69	16 (27.58)
70–79	10 (17.24)
>80	9 (15.52)
Marital status	
Married	57 (98.28)
Single	1 (1.72)
County of residence	
Urban	43 (74.14)
Rural	15 (25.96)

The mean age of the patients was 62.10±13.40 years, with a maximum age of 83 years. The patients’ average BMI was 27.08±4.95 (Table 2).

**Table 2:** The mean of age and BMI of male patients (1992–2023)

Variable	Frequency	Mean±SD	95% CIs	Median (interquartile range)	Min	Max
Age	58	62.10±13.40	58.58–65.63	64.50 (51.00–73.25)	31	83
BMI	31	27.08±4.95	25.26–28.90	27.34 (23.87–30.11)	17.44	42.97

Max, maximum; Min, minimum.

In this study, the right and left breast involvement rate was the same at 48.28% (28 patients) for each side, and only 2 (3.44%) patients had bilateral breast involvement. 55 (94.83%) patients underwent radical mastectomy, 29 (93.55%) out of 31 patients were found to be positive for ER, 25 (83.33%) out of 30 patients were positive for PR, and only 4 (15.39%) out of 26 patients had HER-2 positive. Most patients, 30 (62.50%) out of 58, were in stage 3 of the disease. Additionally, 13 (22.41%) of 58 patients had metastasis, while 4 (6.90%) patients experienced local recurrence. The common sites of metastasis were bone 9 (69.23%), brain 4 (30.77%), lung 3 (23.08%), and liver 1 (7.69%) out of 17 patients, respectively (Table 3).

**Table 3:** Clinicopathological characteristics of male patients with breast cancer (1992–2023)

Variable	Frequency (percentage), *n* (%)
Laterality	
Right	28 (48.28)
Left	28 (48.28)
Bilateral	2 (3.44)
Surgery	
MRM	55 (94.83)
BCS	1 (1.72)
No surgery	2 (3.50)
ER status	
Positive	29 (93.55)
Negative	2 (6.45)
PR status	
Positive	25 (83.33)
Negative	5 (16.67)
HER-2 status	
Positive	4 (15.39)
Negative	22 (84.61)
Stage	
1	4 (8.33)
2	13 (27.09)
3	30 (62.50)
4	1 (2.08)
Local recurrence	
Yes	4 (6.90)
No	54 (93.10)
Metastasis	
Yes	13 (22.41)
No	45 (77.59)
Metastatic sites	
Bone	9 (69.23)
Brain	4 (30.77)
Lung	3 (23.08)
Liver	1 (7.69)

BCS, breast conserving surgery; ER, estrogen receptor; HER-2, human epidermal growth factor receptor-2; MRM, modified radical mastectomy; PR, progesterone receptor.

Table 4 indicates that the mean BMI for patients with metastasis and local recurrence was 25.43 and 21.75, respectively. For patients with high stages, the mean BMI was 27.18. The study found no significant relationship between BMI and the kinds of recurrence and stage of the disease (*P*>0.05).

**Table 4:** Correlation of BMI with metastasis, stage and local recurrence in male patients (1992–2023)

Variable	Variable subgroup	Mean±SD	Difference of mean (95% CIs)	*P*
Metastasis	No	27.40±5.33	1.97 (−3.00 to 6.94)	0.425
	Yes	25.43±1.55		
Stage	1–2	26.37±4.51	−0.81 (−5.50 to 3.58)	0.707
	3–4	27.18±5.52		
Local recurrence	No	27.45±4.85	5.70 (−1.51 to 12.91)	0.117
	Yes	21.75±3.98		

Table 5 shows that most cases of metastasis 6 (66.70%) out of 9 patients and local recurrence 3 (100%) patients occurred in the advanced stages of the disease (stages 3, 4). However, there is no significant relationship between the stage with metastasis and local recurrence (*P*>0.05).

**Table 5:** Correlation of stage with metastasis and local recurrence in male patients (1992–2023)

Variable	Frequency (*N*=48)	Stage 1, 2 (%)	Stage 3, 4 (%)	*P*
Metastasis				
Yes	9	3 (33.30)	6 (66.70)	1.000
No	39	14 (35.90)	25 (64.10)	
Local recurrence				
Yes	3	0	3 (100)	0.543
No	45	17 (37.80)	28 (62.20)	

## Discussion

This study was a retrospective descriptive analysis that included 58 male patients diagnosed with breast cancer during the years 1992–2023, The majority of the patients had a high BMI. Regarding the surgical procedure, the majority of patients received MRM. ER and PR were positive in most cases, and HER-2 was negative in most cases. Most patients were diagnosed in advanced stages 3 and 4 of the disease. Also, metastasis and local recurrence were predominantly observed in these stages. No notable correlation was found between the stage with metastasis and local recurrence, nor between BMI and the kinds of recurrence (metastasis and local).

The study found that the patients had an average age of 62.10 years. The age group most affected was between 60 and 69 years. In the study of Konduri *et al.* ^4^ and the study of Elimimian *et al.* ^30^, The average age of men with breast cancer was about 65 years. Henriques Abreu *et al.* ^31^ in Portugal reported the mean age of patients to be 60 years. Men diagnosed with breast cancer often have an age diagnosis that is ~10 years older than women, who are typically diagnosed beyond the age of 50^15,32^. The research conducted by Brazee *et al.* ^33^ revealed that the mean age of women diagnosed with breast cancer was 55.3 years. The research conducted^34^ revealed that the mean age of women was 49.84 years. The delayed beginning of male breast cancer can be attributed to a combination of factors, including inadequate awareness of early symptoms and the absence of timely detection by mammography. Consequently, men tend to have a longer period of symptoms before receiving a diagnosis compared to women^35^.

Most of the patients in this research were married and lived in urban areas. In Hong *et al.* ^36^‘s study in Korea, 33 (55.9%) out of 59 patients were found to be married. Similarly, in Avila *et al.* ^37^‘s study, 110 (86.6%) out of 127 patients were married. The study conducted by Leone *et al.* ^38^ in the US demonstrated that married patients diagnosed with cancer experienced significantly extended overall survival (OS) in comparison to their single counterparts^38^. In Yadav.‘s study in the US^39^, it was found that 95.4% out of 10 873 patients lived in urban areas. Similarly, in Soni *et al.* ^40^‘s study conducted in India, the majority of patients (67%) out of 106 came from rural regions. The variation in the outcome may be attributed to the disparity in the geographical and cultural context of the research location.

The mean BMI of the participants in the current investigation was 27.08, which aligns with the findings of Soliman *et al.* ^41^‘s study conducted in Egypt. Our study found no significant correlation (*P*>0.05) between BMI with the stage and kinds of recurrence (metastasis and local). However, it was noted that patients with higher stages and those with metastasis tended to have a higher BMI. The alteration in sex hormone levels is a potential mechanism that may explain the correlation between weight fluctuations and the susceptibility to breast cancer in males. Obesity in men is linked to elevated estrogen levels and reduced levels of testosterone and sex hormone-binding globulin. This results in increased availability of estrogen^42,43^.

On the other hand, variable results have been seen in different studies, so this study reported the involvement of both sides of the breast as the same (48.28%). In the research by Hong *et al.* ^36^, it was shown that 31 (54.4%) patients exhibited a greater prevalence of left-side involvement. Similarly, in the study of Rolf *et al.* ^44^, the left breast was more involved in 32 (45%) patients. While Soni *et al.* ^40^ with a rate of 52.8% and Chikaraddi *et al.* ^45^ with 61.5% have reported more right-side involvement.

Nearly the majority of patients (94.8%) in our research received MRM surgery, which is consistent with findings from earlier studies in this field^31,36,39,44^. The function of BCS in men is less extensive compared to women. In men, BCS is typically preferred at the initial stages of cancer, when it is possible to obtain sufficient removal of the tumor margins. Attaining this goal is challenging owing to the relatively tiny size of male breasts^46^.

The present study’s findings indicate that most male participants showed positive expression of ER and PR, while they showed negative expression of HER-2. In the study conducted by Eggemann *et al.* ^47^, the majority of patients exhibited a positive expression of ER at a rate of 97.4% and PR at a rate of 92.9%. HER-2 overexpression was only seen in 14.5% of patients. The study conducted by Soni. and colleagues found that 48% of patients showed positive expression of ER, 25% showed positive expression of PR, and 43.4% showed negative expression of HER-2. The decreased expression of ER/PR in their study can be attributed to the absence of hormone receptor testing facilities in prior years^40^. Patients with positive ER and PR statuses have demonstrated improved survival rates. However, more validation is required to establish the prognostic significance of these biomarkers^26^.

Most of the patients (64.58%) in this research were diagnosed in stages 3 and 4. This finding is consistent with previous studies^45,48^. However, according to the research conducted by Avila *et al.* ^37^, 76.3% of the patients were classified as stage 1 or 2. In a research by Tahmasebi and colleagues in southern Iran, it was found that 74.5% of patients were diagnosed with stage 1. This finding contradicts the results of our study. This difference in the results may be attributed to inadequate awareness of the initial signs of breast cancer or delayed patient referral in some regions^28^. The advanced stage at the time of diagnosis is responsible for the decreased survival rate in males compared to females^15^. In this study, most metastasis cases and all local recurrence cases occurred in the advanced stages of the disease. The incidence of metastasis was 22.41%, and the occurrence of local recurrence was 6.9%. In Tahmasebi *et al.* ^23^‘s study, the incidence of metastasis was 15.5%, whereas in Soni *et al.* ^40^‘s study, this rate was seen to be 19.8% of patients.

The predominant metastatic sites in this study were bone, brain, lung, and liver, respectively. In contrast to previous studies indicating lower brain metastasis rates^18,31^, our study showed that the brain (30.77%) is the common site of metastasis after the bone (69.23%). Similarly, a study in Turkey^27^ found that metastasis to the brain (21.4%) is the third most prevalent, behind bone and brain metastasis. The difference may be attributed to the limited sample size in Turkey’s study (41 patients) and our study (58 patients).

This study had some limitations. Breast cancer in men is rare, so the sample size was also small, which caused a disturbance in the interpretation of some results. Also, due to the almost long-term trend, much data related to the patients was unavailable in older cases, and the patients’ files were not well completed. In this study, the absence of a significant statistical relationship in examining the relationships of important variables may be due to the study’s small sample size, so it is suggested that future studies with larger sample sizes and multicenter studies be conducted, in other hand given that a census method was employed in this study and all eligible patients during the study years were included, the sample size remains very small. This limited sample size is insufficient to meet the analytical objectives of conducting multivariate regression analysis. Furthermore, for multivariate regression analysis, in addition to the initial sample size calculation, it is recommended to add ~20 samples for each variable included in the analysis. Therefore, multivariate analyses were not feasible, and the study primarily focuses on the descriptive aspects of the results

## Conclusion

Most of the cases of male breast cancer occurred at an advanced age, and the diagnosis was in the advanced stages of the disease, which is the reason for late diagnosis and the occurrence of advanced stages of the disease in men and probably reduces their survival. Currently, breast cancer is considered a health problem in Iran and the world, and due to its lower incidence in men, the sensitivity to it in society is less. Moreover guidance of at-risk groups to cooperate in screening and timely diagnosis so the disease can be treated quickly. Nevertheless, this study establishes the foundation for future studies about male breast cancer. This field should facilitate the management of a larger patient population, enhance comprehension of this disease in males, identify its distinct risk factors, and differentiate it from breast cancer in females. Consequently, it will enable the selection of suitable screening and treatment modalities.

## Ethical approval

This study was carried out after the approval of the ethics committee of Babol University of Medical Sciences in such a way that all steps of the Declaration of Helsinki were observed.

## Consent

To observe ethical considerations, in this study, issues such as obtaining an ethics code with the number IR.MUBABOL.REC.1400.140, maintaining confidentiality of information at all stages were respected. Given that the data collected was of a secondary nature and did not involve human sampling, the requirement for obtaining informed consent did not apply. This determination was confirmed by the Ethics Committee of Babul University of medical science. we referred to the medical records archive of the hospital. The work has been reported in line with the Strengthening the Reporting of Cohort Studies in Surgery criteria.

## Source of funding

Not applicable.

## Author contribution

M.A.J., D.M., H.A.N. and A.B. were the principal investigators and designed the study. M.A. searched literature. M.A.J. and D.M. supported the interview development. M.A. and H.M. collected data and prepared data for qualitative analyses. M.A.J. and A.B. supervised data collection. H.A.N. and A.B. analyzed data. M.A. drafted the manuscript and both M.A.J. and D.M. supported drafting the manuscript. All authors have provided comments and critical revisions to the manuscript. All authors approved the final manuscript prior to submission.

## Conflicts of interest disclosure

The authors declare no conflicts of interest.

## Research registration unique identifying number (UIN)

Approval and registration of the proposal in the Babul University Research Council and then obtaining the ethics code number (IR.MUBABOL.REC.1400.140.).

## Guarantor

Mohammad-Ali Jahani.

## Data availability statement

The datasets used and/or analyzed during the current study are available from the corresponding author on reasonable request.

## Provenance and peer review

Our paper wasn't invited.
